# Foundations of Livestock Behavioral Recognition: Ethogram Analysis of Behavioral Definitions and Its Practices in Multimodal Large Language Models

**DOI:** 10.3390/ani15203030

**Published:** 2025-10-19

**Authors:** Siling Zhou, Wenjie Li, Mengting Zhou, Ryan N. Dilger, Isabella C. F. S. Condotta, Zhonghong Wu, Xiangfang Tang, Yiqi Wu, Tao Wang, Jiangong Li

**Affiliations:** 1 State Key Laboratory of Animal Nutrition and Feeding, College of Animal Science and Technology, China Agricultural University, 2 W Yuanmingyuan Ave, Beijing 100193, China; zhousiling@cau.edu.cn (S.Z.);; 2 State Key Laboratory of Animal Nutrition and Feeding, Institute of Animal Sciences, Chinese Academy of Agricultural Sciences, 2 W Yuanmingyuan Ave, Beijing 100193, China; 3 Department of Animal Sciences, University of Illinois at Urbana-Champaign, 1207 W. Gregory Dr., Urbana, IL 61801, USAicfsc@illinois.edu (I.C.F.S.C.); 4 AniEye Technology Co., Ltd., Hangzhou 311215, China; wgxing0330@mumutech.co

**Keywords:** ethogram, computer vision, language, animal welfare, precision livestock farming

## Abstract

**Simple Summary:**

Accurate behavior monitoring is critical for livestock health, welfare, and productivity. While computer vision is widely used for automated tracking, inconsistent behavior definitions across studies can reduce the reliability, consistency, and interpretability of data annotation and model performance. In this study, we used natural language processing (NLP) to examine 655 behavior definitions from research involving cattle, pigs, sheep, and horses. We identified common linguistic patterns in how behaviors are described. To assess the practical value of these patterns, we incorporated them into a behavior recognition task using a large language model (LLM). The results showed that the LLM performed more consistently and accurately when guided by these language patterns. Our findings suggest that improving the clarity and consistency of behavior definitions can enhance the reliability and interpretability of AI-based livestock behavior monitoring.

**Abstract:**

Computer vision offers a promising approach to automating the observation of animal behavior, thereby contributing to improved animal welfare and precision livestock management. However, the absence of standardized behavioral definitions limits the accuracy and generalizability of artificial intelligence models used for behavior recognition. This study applied natural language processing techniques to analyze 655 behavior definitions related to feeding, drinking, resting, and moving, as reported in the livestock research literature published between 2000 and 2023. Clustering and structural analyses revealed consistent semantic patterns across behavior categories. Feeding and drinking behaviors were concisely defined in 6–10 words, including the semantic elements of body parts, actions, and action objects. Resting and moving behaviors were described in 6–15 words. Resting behavior was defined by actions and action objects, while moving behaviors were characterized by action words only. By integrating these structured definitions into prompts, ChatGPT-4o achieved an average correspondence score of 4.53 out of 5 in an image-based piglet behavior annotation task. These findings highlight the value of standardized behavior definitions in supporting more accurate and generalizable behavior recognition models for precision livestock farming.

## 1. Introduction

With advancements in animal surveillance systems and computer vision algorithms, non-invasive animal monitoring and automatic behavioral analysis have the potential to assist in reducing the workload for animal caretakers [1,2,3,4]. Researchers in agricultural engineering have applied deep learning models, originally developed for other purposes, to animal behavior recognition tasks. YOLOv5 has been used to recognize dairy cow behaviors, introducing indices such as the Comfort Index (CI), Stall-Use Index (SUI), and Cow Stress Index (CSI) to assess heat stress in cattle [5]. Mask R-CNN has been applied to identify group-housed pigs’ feeding behavior by analyzing the occupancy of the feeding area [6]. A two-stage deep learning method has also been proposed to recognize sheep behaviors, including both normal activities and destructive behaviors like biting and climbing [7]. While these systems show potential, they remain largely experimental and have not yet been widely adopted in routine husbandry practices, limiting their practical application in real-world settings. Moreover, animal behavior recognition tasks (e.g., classification, detection, quantification) continue to face scientific skepticism, not due to the technical feasibility of identifying behaviors, but because of their limited interpretability, insufficient standardization, and weak integration with established ethological frameworks. Unlike traditional ethological approaches that emphasize context-rich descriptions and theoretically grounded classifications, many automated methods focus on quantifying posture, movement, and spatial features [8,9,10]. While effective at detecting behavioral patterns, such approaches often lack the theoretical grounding that links observed behavior to underlying physiological states or adaptive functions, limiting their explanatory value in behavioral science [11,12].

Scientists typically describe behaviors with text labels and natural language definitions [13]. These traditional methods have laid a valuable foundation for behavior annotation in animal science, offering biologically meaningful insights through expert-defined ethograms and observational descriptions. Behavior definitions serve as the basis for interpreting animal actions. However, observers are subjective when interpreting definitions, depending on their background knowledge and experience. This subjectivity presents challenges for automated and objective behavior recognition and scientific analysis [14,15]. Although there is no universal standardized method for developing an animal behavior ethogram, researchers in animal behavior studies generally follow several scientific conventions to ensure consistency and comparability [16]. A well-constructed ethogram is expected to meet essential criteria such as interpretability, objectivity, and specificity, allowing it to serve as a reliable framework for behavioral observation and classification [17]. Typically, an ethogram consists of two components: behavior labels and behavior descriptions. The behavior label is a concise term that distinguishes one behavioral category from another and carries important semantic characteristics [18,19]. The behavior description provides further detail to guide accurate observation and classification, typically incorporating standardized terminology and relevant contextual elements such as body parts, postures, or types of movement [20,21,22,23].

In traditional behavioral research, to ensure the consistency and reliability of manual annotations, researchers commonly employ inter-observer reliability (IOR) and intra-observer reliability assessments [24,25]. This statistical approach quantifies the agreement between multiple observers and helps reduce subjectivity and individual bias, thereby enhancing the overall objectivity of behavioral data [13]. However, even with IOR procedures in place, manual annotation remains a time-consuming and labor-intensive process, particularly in large-scale or long-duration studies. Observer bias may still arise from differences in observational perspective, prior experience, or specific research goals. Current animal behavior recognition algorithms heavily depend on such manual annotations, which not only demand significant human effort but are also sensitive to the variability introduced by observational conditions and study contexts [11,26]. Automating the recognition process through precise and standardized behavioral definitions has the potential to improve objectivity and reduce annotation workload [26,27]. However, the lack of universally standardized definitions continues to hinder consistent and accurate behavior classification, as semantically similar descriptions may be interpreted or labeled differently across observers or research settings.

Recently, scholars have raised concerns about subjectivity in traditional animal behavior research, which affects the objectivity of intelligent behavior recognition systems [2,11]. Behavior definitions, as linguistic descriptions derived from human observation of visual features, lie at the intersection of vision and language research. A key concept in this domain is semantic correspondence, which refers to the ability to consistently align elements in visual data (e.g., objects, movements, spatial cues) with their semantic descriptions in natural language [28]. For example, the system should recognize that a cow lying down under different lighting or from various camera angles still corresponds to the same behavioral label, such as “lying.” This alignment is essential for behavior recognition tasks that require interpreting visual events through linguistic definitions. However, current multimodal large language models still struggle with achieving robust semantic correspondence in dynamic and complex video contexts, especially for fine-grained animal behaviors [29]. Therefore, establishing a scientific, unified, and applicable system for animal behavior definitions is significant for advancing animal behavior science and artificial intelligence research.

To address the current status of semantically defined definitions in livestock research, this study analyzes livestock behavior ethograms using natural language processing techniques and manual semantic analysis. The goal is to improve the clarity and consistency of behavior definitions by identifying underlying semantic patterns across common behavior categories. These patterns have the potential to enhance the interpretability and transparency of behavior recognition tasks by focusing on key semantic features, similar to the strengths of traditional ethological analysis, which emphasizes clear and consistent definitions for better understanding and interpretation of animal behaviors.

## 2. Methods

### 2.1. Data Collection

The target papers were searched with the keyword “ethogram” published between 2000 and 2023 in three journals: *Applied Animal Behavior Science*, *Animal Behavior*, and *Journal of Animal Science*. Only papers that included an ethogram table with behavior categories and corresponding behavior definitions were included in the analysis, while studies that mentioned “ethogram” but lacked a detailed behavior classification table were excluded. A total of 417 relevant papers were collected. The selected papers covered species such as cattle, sheep, pigs, horses, chickens, ducks, geese, rabbits, deer, cats, and lions. From each paper, information on animal species, behavior labels, and behavior definitions was extracted and compiled into a behavior definition table.

**Figure 1:**
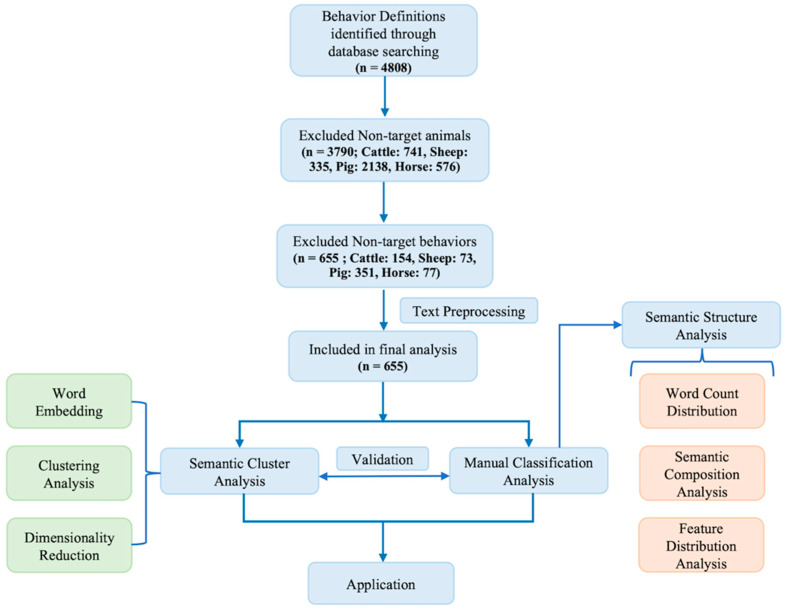
Overview of the data selection and analysis process (n = number of behavior definitions).

To refine the dataset, we focused on four major livestock species: cattle, sheep, pigs, and horses. These species were selected for their economic significance, frequent use in animal behavior and welfare research, as well as their shared characteristics as quadrupedal terrestrial mammals. Species such as poultry were excluded due to their distinct physiological traits, including bipedal locomotion and other behaviors that complicate the comparison of behavior patterns across species. These differences complicate the comparison of behavior patterns across species. From the selected literature, a total of 3790 behavior definitions were extracted. Among these, behaviors related to eating, drinking, resting, and moving were further selected, as these fundamental activities are closely linked to animal survival, growth, and welfare. Behavior classification was based on the semantic interpretation of the behavior labels provided in the ethograms. For example, motionless postures such as lying or standing still were categorized as resting, while actions such as walking, running, or jumping were classified as moving. Social behaviors were excluded due to the semantic ambiguity in definition and the difficulty of achieving consistent classification across species. As a result, 655 behavior definitions were retained to construct the final behavior definition dataset (Figure 1).

### 2.2. Data Preprocessing

The preprocessing stage is a critical step in preparing the text for analysis. In this phase, Natural Language Processing (NLP) techniques were applied to clean and standardize the behavior definitions, ensuring they were in an optimal format for subsequent analysis. NLP is a field of artificial intelligence that focuses on enabling computers to understand, interpret, and generate human language [30]. It provides tools such as tokenization, stopword removal, and lemmatization, which are essential for extracting meaningful patterns from unstructured text data.

Initially, commonly occurring words and special characters were removed from the behavior definitions [31]. Elements such as articles, conjunctions, and punctuation marks often lack significant meaning and may introduce noise into the dataset. Lemmatization techniques were applied to standardize inflected word forms, ensuring semantic consistency across sentences [32]. For instance, variations such as “walking,” “walks,” and “walked” were reduced to their base form, “walk,” preserving their shared meaning. Lemmatization only addressed morphological variations, and semantically related or synonymous terms (e.g., “resting” and “lying”) were retained as distinct expressions during preprocessing. To focus on behavior-related content, animal-specific terms (e.g., “pig,” “cow,” and “sheep”) were excluded from the dataset [33]. This step provided a more accurate and consistent textual foundation for subsequent behavior analysis. Upon completing these preprocessing steps, a dataset comprising 655 cleaned and standardized behavior definitions was constructed.

### 2.3. Semantic Cluster Analysis of Behavior Definitions

Following text preprocessing, behavior definitions in the dataset were vectorized using the Word2Vec model, which transforms textual descriptions into numerical vectors suitable for clustering analysis (Equation (1)). Word2Vec is a neural network-based model that generates word embeddings by analyzing the context of words, placing semantically similar words nearby within a vector space [34]. Using the Word2Vec implementation in the Gensim library, each word in the behavior definitions was mapped to a 100-dimensional vector space. To represent each behavior definition as a sentence vector, the average of the word vectors corresponding to its constituent words was computed.

(1) $$\begin{array}{c} v_{d}=\frac{1}{N}\overset{N}{\underset{i=1}{\sum }}v_{i}\; \end{array}$$

(2) $$\begin{array}{c} P\left(w_{t}|w_{t-1},w_{t+1}\right)=\frac{exp\left(v{\left(w_{t}\right)}^{⊤}v_{context}\right)}{\sum_{w\in V}exp\left(v{\left(w\right)}^{⊤}v_{context}\right)} \end{array}$$

where, $v_{d}$ represents the vector of the behavior definition d, $v_{i}$ represents the vector of the i-th word in d, N is the number of words in d, $P\left(w_{t}|w_{t-1},w_{t+1}\right)$ is the probability of the word $w_{t}$ given its surrounding context words $w_{t-1}$ and $w_{t+1}$, and $v_{context}$ represents the context vector.

The K-means clustering algorithm was used to analyze the generated word vectors. K-means is an unsupervised method that partitions data into clusters by minimizing intra-cluster variance [35]. It is well-suited for vector spaces such as those produced by Word2Vec, where terms are represented as vectors [36]. The optimal number of clusters (k) was determined by testing values from 1 to 8, using the elbow method to identify the point of diminishing returns and the silhouette score to assess cluster cohesion. Principal Component Analysis (PCA) was applied to reduce the dimensionality of the word vectors [37], projecting the data onto two components that capture the greatest variance for visualization. The word vectors were mean-centered before PCA to ensure accurate component extraction.

All text processing, vector modeling, clustering, and visualization procedures were implemented in Python 3.8 with the functional programming packages, including nltk for preprocessing, gensim for Word2Vec training, scikit-learn for clustering and dimensionality reduction, and matplotlib for visualization.

### 2.4. Validation of Cluster Results Through Classification Analysis

To validate the clustering results and address the limitations of automatic methods in handling ambiguous or overlapping behaviors, 655 behavior definitions were manually classified into three categories: Eating and Drinking, Resting, and Moving, based on the classification criteria outlined in Table 1. This classification was based on the two-cluster solution from Section 2.3, which showed the highest internal validity, and the three-cluster solution as the next-best structure. The three-category classification was chosen to allow for finer semantic analysis while aligning with functional distinctions commonly used in ethological research. Eating and Drinking behaviors were merged due to their semantic proximity as identified in the clustering analysis. Each behavior definition was assigned to one category based on its primary focus. For behaviors involving multiple actions (e.g., “eating while moving”), classification was based on the behavior most central to the description’s functional intent. All classifications were conducted by a single researcher in accordance with the predefined criteria to ensure internal consistency and reproducibility throughout the labeling process.

A total of 233 behaviors were classified as Eating and Drinking, 257 as Resting, and 165 as Moving. The Eating and Drinking category includes behaviors like “eat,” “drink,” “graze,” and “forage.” Resting behaviors involve stillness, relaxation, or low energy expenditure, such as lying, standing still, and sleeping. Moving behaviors encompass locomotion or active engagement, like walking, running, jumping, and exploring, which are critical for survival and reproduction.

### 2.5. Semantic Structure Analysis and Validation of Behavioral Categories

Building on the manually classified behavior categories, a two-part analysis was conducted to examine how animal behaviors are described in ethological datasets and to identify structural patterns and dominant semantic elements in existing behavior definitions. This analysis focused on extracting recurring linguistic features and descriptive structures across categories, providing insights into prevailing annotation practices and potential directions for ethogram standardization. In addition, to assess the consistency of the manual classification, the predefined categories were compared with the groupings produced by unsupervised clustering.

In the first part, three linguistic features were examined: word count distribution, semantic element composition, and keyword extraction. Word count served as a proxy for descriptive complexity, with longer definitions reflecting more nuanced behaviors. Semantic element analysis identified functional building blocks in behavior descriptions, while keyword extraction highlighted prominent lexical elements characterizing each category.

In the second part, behavior definitions were vectorized using the terms frequency-inverse document frequency (TF-IDF) and Word2Vec to capture both lexical salience and semantic context. TF-IDF was used to quantify the importance of individual terms within and across definitions based on their distribution patterns [38], while Word2Vec enabled the representation of words in a continuous vector space based on co-occurrence, capturing semantic relationships beyond exact word matching [34]. These approaches allowed for a robust comparison of clustering outcomes with the manual classification framework.

Firstly, word count distribution was analyzed for behavior definitions in each manually classified behavior category. Definitions were grouped into six intervals based on length: 1–5, 6–10, 11–15, 16–20, 21–25, and 26 or more words. This analysis aimed to quantify the descriptive length of behavior definitions and to compare structural variation in expression across categories.

Next, semantic element composition was examined by decomposing each definition into seven components: Subject, Body Parts, Action or Verb, Location or Object, Time-related Description, Action-related Description, and Distance-related Description. These components were derived inductively from common syntactic roles and functional patterns observed in the data. The full classification scheme is summarized in Table 2.

Following this, keyword extraction and visualization were performed based on the distribution of semantic elements. For each behavior category, if a specific semantic element, such as Action or Verb, Body Parts, Location or Object was present in more than 50% of the definitions, it was considered representative of that category. Relevant lexical items associated with each representative element were aggregated from the corresponding definitions, and word clouds were generated accordingly.

Finally, the appearances of each semantic group in behavior definitions were calculated as shown in Equation (3).

(3) $$\begin{array}{c} f\left(E,C\right)=\frac{n\left(E,C\right)}{N\left(C\right)}\times 100\% \end{array}$$

where, $f\left(E,C\right)$ represents the frequency of the semantic element E in the behavior category C, $n\left(E,C\right)$ is the number of occurrences of E in C, and $N\left(C\right)$ is the total number of behavior definitions in C.

To contrast the semantic representations of behavior definitions under the framework of manual classification, each definition was first transformed into a numerical vector using two natural language processing techniques: TF-IDF (Equation (4)) and Word2Vec (Equation (1)). TF-IDF represents a document as a sparse vector, where each dimension corresponds to a term weighted by its frequency in the document and its inverse frequency across the corpus, thereby highlighting terms with high discriminative value [39]. In contrast, Word2Vec generates dense, continuous word embeddings based on distributional co-occurrence patterns in large corpora, capturing contextual semantic relationships between words [40]. For each definition, the embeddings of individual words were averaged to obtain a single vector representation.

(4) $$\begin{array}{c} v{\left(d\right)}_{j}=TF\left(t_{j},d\right)\cdot IDF\left(t_{j}\right) \end{array}$$

(5) $$\begin{array}{c} TF\left(t_{j},d\right)=\frac{f_{t_{j},d}}{\sum_{k}f_{k,d}} \end{array}$$

(6) $$\begin{array}{c} IDF\left(t_{j}\right)=log\left(\frac{N}{1+n_{t_{j}}}\right) \end{array}$$

where, $v{\left(d\right)}_{j}$ represents the j-th component of the vector for the behavior definition d, $TF\left(t_{j},d\right)$ is the term frequency of the term $t_{j}$ in d, $IDF\left(t_{j}\right)$ is the inverse document frequency of $t_{j}$, $f_{t_{j},d}$ is the frequency of term $t_{j}$ in d, $\sum_{k}f_{k,d}$ is the total frequency of all terms in d, N is the total number of behavior definitions, and $n_{t_{j}}$ is the number of behavior definitions containing term $t_{j}$.

Following vectorization, the resulting semantic embeddings of behavior definitions were used as input for unsupervised clustering in the vector space, where proximity between vectors reflects underlying semantic similarity. To further quantify these relationships, pairwise cosine similarity between vector representations was computed (Equation (7)), serving as a metric for semantic proximity in subsequent comparative analysis. Manual classification results served as reference labels to examine the correspondence between predefined categories and clusters derived from the vector-based representations. This comparison was intended to assess the consistency between human-defined and data-driven classifications and to identify potential areas of semantic ambiguity or category overlap within the existing taxonomy.

(7) $$\begin{array}{c} cos\left(v_{1},v_{2}\right)=\frac{\sum_{i=1}^{d}v_{1,i}v_{2,i}}{\sqrt{\sum_{i=1}^{d}v_{1,i}^{2}}\sqrt{\sum_{i=1}^{d}v_{2,i}^{2}}} \end{array}$$

where, $cos\left(v_{1},v_{2}\right)$ represents the cosine similarity between vectors $v_{1}$ and $v_{2}$, $v_{1,i}$ and $v_{2,i}$ are the i-th components of these vectors, and d is the dimension of the vectors.

## 3. Results and Discussions

### 3.1. Semantic Cluster Analysis

**Figure 2:**
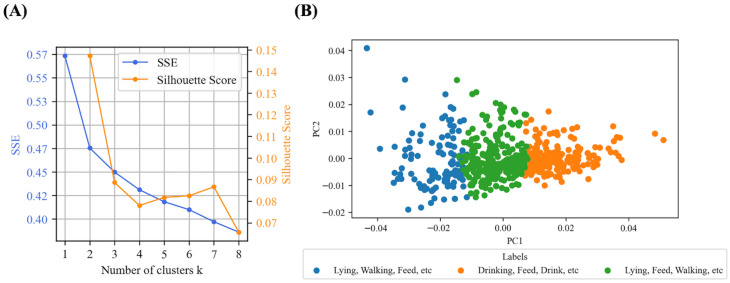
Clustering analysis based on Word2Vec vectorization. (**A**) Cluster evaluation using Sum of Squared Errors (SSE), which measures within-cluster compactness (lower is better), and Silhouette Score, which evaluates between-cluster separation (higher is better); (**B**) PCA visualization of clustered behavior definitions using the first two principal components. Cluster labels indicate the three most frequent behavior labels associated with the definitions in each cluster, automatically extracted after clustering.

The K-means clustering algorithm was applied to the vectorized data, and clustering performance was evaluated using both the Elbow Method and the Silhouette Score [41]. The Elbow Method is commonly used in cluster analysis to plot the sum of squared errors (SSE) against the number of clusters, identifying the point where the decrease in SSE levels off. This “elbow” point reflects a trade-off between model complexity and clustering quality. As shown in Figure 2A, the SSE decreases consistently as the number of clusters increases, but no clear elbow point is observed. A slightly slower rate of decline is visible around k = 3, which may indicate a point where additional clusters yield relatively smaller improvements. However, in the absence of a distinct turning point, this remains an interpretative judgment rather than a definitive conclusion. In parallel, the Silhouette Score, which ranges from −1 to 1, was used to assess the cohesion within clusters and their separation. Higher values indicate better-defined clusters, but in this case, the score reached its highest value at k = 2 and gradually declined as the number of clusters increased. While the result at k = 2 suggests stronger internal consistency, k = 3 was selected in order to explore whether finer subdivisions of behavior definitions might emerge. It is important to note, however, that the silhouette scores remain low overall, indicating that the clusters are not clearly separated in the semantic space defined by the data.

As shown in Figure 2B, the clustering results are visualized using principal component analysis (PCA), which projects the high-dimensional semantic data onto a two-dimensional space. Each point represents a behavior definition, with its color indicating cluster assignment. The label descriptions in the figure legend were automatically generated using Latent Dirichlet Allocation (LDA), a generative probabilistic model that identifies latent topic structures in a collection of texts based on word co-occurrence patterns [42]. The LDA-generated labels reveal that certain behaviors, such as “Feeding,” “Lying,” and “Walking,” appear in multiple clusters. Specifically, “Feeding” is found across all three groups, while “Lying” and “Walking” are shared by at least two. This overlap indicates significant semantic ambiguity among behavior definitions, suggesting that the clusters are not clearly separated. For instance, “Feeding” can describe both passive activities, like eating while resting, and active behaviors, such as grazing while moving. The contextual variability of these definitions complicates the clustering process. This challenge may be exacerbated by information loss during the vectorization of behavior definitions, which can prevent a more nuanced representation of these behaviors. The diversity in how behaviors are described across different contexts and interpretations further complicates the identification of distinct clusters. This limitation suggests that alternative methods or finer granularity may be needed to better capture the complexity of behavior definitions.

The overlap of “Lying” and “Walking” across clusters may be partly attributed to the preprocessing stage, particularly the use of lemmatization, which standardizes inflected forms into a single base form. While this process improves lexical consistency across definitions, it may also obscure context-specific distinctions by collapsing semantically nuanced expressions into the same lexical representation. In addition, some behaviors are similar in terms of the body parts involved; for instance, both lying down and walking engage the limbs. These physiological and functional commonalities may result in similar textual descriptions, leading to proximity in the semantic embedding space and contributing to their co-occurrence across clusters. For example, in Figure 2B, the behaviors Lying and Walking co-occur in the LDA-generated labels on two occasions, suggesting that the model has difficulty distinguishing between them due to shared linguistic features in their definitions.

These findings indicate that the clustering results do not reflect clearly separated behavioral categories, but instead correspond to areas of similarity in the vectorized feature space. This similarity is shaped by shared vocabulary and definitional ambiguity rather than clear functional distinctions. Although PCA improves interpretability by enabling visual inspection of cluster distribution, it does not address the limitations of the input vectors derived from textual behavior definitions.

A key challenge arises when some behavior definitions contain elements associated with multiple categories. For example, the definition “animals grazing with their head down can be stationary or moving ≤ 3 consecutive steps” includes terms related to both “grazing” and “moving.” As a result, its vector representation may fall between these two clusters in the feature space. This does not indicate the presence of a distinct behavioral type, but rather reflects semantic overlap caused by shared linguistic features. Such cases highlight the limitations of unsupervised clustering and the importance of expert review. Automated methods alone may struggle to resolve ambiguities that stem from the way behaviors are described, especially when textual definitions do not align neatly with functional or ethological categories. Therefore, expert validation remains essential for verifying clustering outputs and refining behavior classification frameworks.

### 3.2. Semantic Structure Analysis

#### 3.2.1. Word Count Distribution

**Figure 3:**
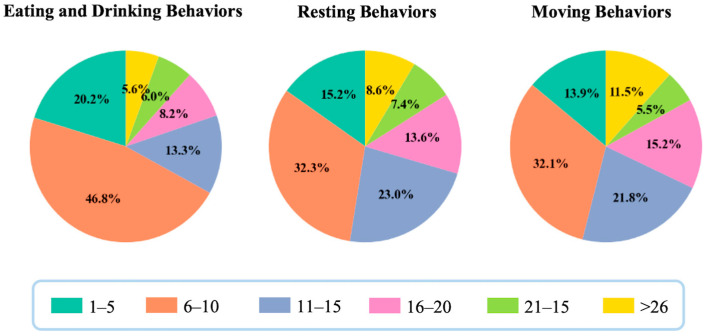
Distribution of word count in behavior definitions.

To evaluate the descriptive complexity of behavior definitions, we analyzed the distribution of word counts across the three manually classified behavior categories, as shown in Figure 3. Among eating and drinking behaviors, 80.3% of the definitions were composed of 1–15 words, suggesting that these behaviors are generally described using concise and focused language, likely due to their relatively consistent structure and clear functional boundaries. In contrast, 84.1% of resting behavior definitions and 83.0% of moving behavior definitions fell within the range of 1–20 words, reflecting greater variation in linguistic structure. This broader range may be attributed to the need to describe diverse postures, durations, contexts, or directional movements. In this analysis, we focused on the word-count intervals that, starting from the most frequent, cumulatively accounted for approximately 80% of the definitions in each behavior category. This frequency-based selection strategy allowed us to capture the predominant patterns in definition length while minimizing the influence of exceptionally short or long entries. By concentrating on the most typical segment of each category, we enabled clearer comparisons of linguistic complexity and explored how variation in expression may reflect underlying behavioral diversity.

#### 3.2.2. Semantic Structure Composition and Keywords Extraction

**Figure 4:**
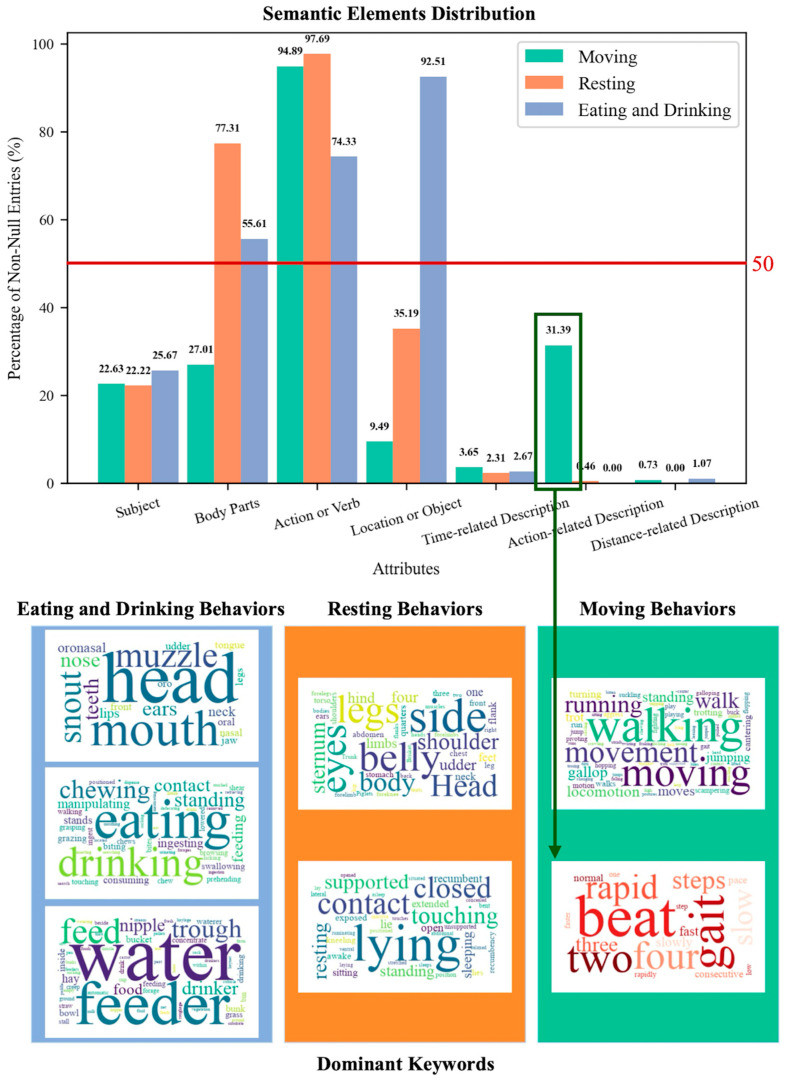
Semantic structure and lexical content of behavior definitions. The bar chart shows the proportion of behavior definitions in each category that contain a given semantic element. The numbers above the bars indicate the percentage of definitions that include that element. The word clouds below display the most frequent terms extracted from selected semantic fields, with font size reflecting term frequency.

To identify prominent semantic elements within each behavior category, we examined the proportion of definitions containing each semantic element, as shown on the y-axis of Figure 4. Elements that appeared in more than 50% of definitions were considered core structural features of that category. In the Eating and Drinking category, the element Action or Verb appeared in 74.33% of definitions, Body Parts in 55.61%, and Location or Object in 92.51%. These three elements together specify the action performed, the body part involved, and the spatial context, resulting in a more comprehensive and informative behavior definition. In contrast, definitions in the Resting Behavior category were dominated by Action or Verb, which appeared in 97.69% of the cases, followed by Body Parts in 77.31%. Definitions of Moving Behaviors contained fewer semantic elements overall, with 74.33% including only the Action or Verb element. However, Action-related Descriptions, including elements such as intensity and direction, were found in 31.39% of the moving behavior definitions, indicating the importance of these parameters for differentiating between dynamic motor activities.

To explore the lexical content of these dominant elements, word clouds were generated based on terms aggregated from elements that met the representativeness threshold in each category. In the Eating and Drinking category, terms such as “head,” “mouth,” “eating,” “drinking,” “feeder,” and “water” appeared with high frequency, reflecting the anatomical and environmental context central to feeding behaviors. In Resting Behaviors, the most salient terms included “belly,” “side,” “lying,” and “contact,” which emphasize bodily posture and spatial configuration. Moving behaviors were primarily characterized by terms such as “moving,” “walking,” and “running.” Additionally, terms like “gait,” “steps,” “two,” “four,” and “rapid” were frequently observed in the Action-related Description element, describing variations in rhythm, directionality, and movement style.

Through an in-depth analysis of the semantic structure, it can be observed that while the definitions of eating and drinking behaviors are relatively concise, they require a higher level of detail, involving the simultaneous presence of body parts, actions, and objects. In contrast, the definitions of resting and moving behaviors, although lengthier, require fewer elements. It is important to note that while eating and drinking behaviors necessitate a higher level of completeness in their elements, this reflects the significance and complexity of these behaviors in animal life. When animals engage in eating and drinking, specific body parts such as the head and mouth, and actions such as eating and drinking are involved, along with clear objects of the action, such as a feeder or water trough. These elements provide a comprehensive and concrete description of the behavior. On the other hand, although resting and moving behaviors involve fewer semantic components, their complexity must still be considered during recognition and classification. Resting behaviors primarily rely on the Action or Verb component to describe stationary states, but must also account for postural variation, such as lying on the side or stomach. Likewise, in moving behaviors, Action or Verb remains the core descriptor, but parameters such as intensity, speed, and spatial orientation are essential for distinguishing between different movement types such as walking, trotting, or running. Consequently, moving behavior descriptions often incorporate additional elements such as duration, locomotor intensity, and relative distance.

These findings underscore the importance of considering both the quantity and type of semantic components in behavior definitions when developing ethogram-based recognition systems. Rather than relying solely on core action verbs, effective classification should incorporate structured descriptors such as temporal cues, intensity, and spatial context. These elements are essential for capturing the complexity of behaviors and distinguishing subtle variations. Continued research into the semantic structure of behavior definitions can support more consistent and biologically meaningful annotation practices.

### 3.3. Consistency Between Manual Classification and Semantic Clustering

**Figure 5:**
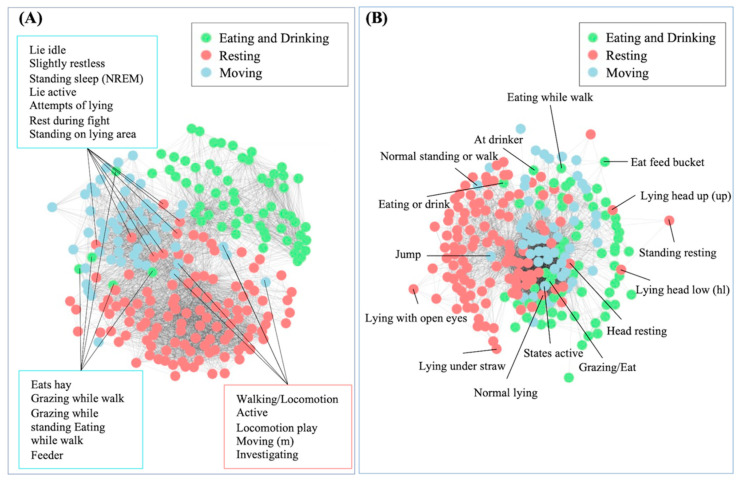
Semantic networks of behavior definitions based on TF-IDF and Word2Vec representations. Each node represents one behavior definition, and edges indicate pairwise cosine similarity, where shorter distances reflect greater semantic similarity. (**A**) Network based on TF-IDF vectors; (**B**) Network based on Word2Vec embeddings. Node colors correspond to manually assigned behavior categories. Central behaviors tend to use general terms, resulting in higher semantic similarity with other behaviors, while more specific or compound behaviors are found at the outer layers.

To assess the alignment between manual classification and automated semantic clustering, behavior definitions were vectorized using two approaches: TF-IDF and Word2Vec. Figure 5 presents the resulting semantic similarity networks, where each node represents a behavior definition and the distance between nodes reflects their cosine similarity. Node colors correspond to the manual classification: green for Eating and Drinking, red for Resting, and blue for Moving. Compared to Word2Vec, the TF-IDF-based network produced using the term frequency-inverse document frequency shows clearer separation into three clusters that broadly correspond to the manually assigned categories. This reflects the method’s reliance on distinctive word choices that are more easily distinguishable between categories. In contrast, the Word2Vec-based network displays a more continuous distribution, with overlapping clusters and weaker boundaries. Since Word2Vec is based on patterns of word co-occurrence in similar contexts, it tends to group together definitions that contain similar vocabulary or appear in related linguistic environments, even if they belong to different manually defined categories.

Further inspection of the Word2Vec-based network revealed a three-layer radial structure. As shown in Figure 5B, behaviors near the center, such as Grazing and Normal lying, tended to use common, non-specific terms like “standing,” “moving,” and “head,” which caused them to be more semantically similar to a broad range of other behaviors. In contrast, moderately specific behaviors such as “Head resting” and “Jump” were placed around the center, while more specific or compound behaviors, such as “Lying head up,” “Lying head low,” and “Eating while walking,” appeared at the outermost layer. This distribution suggests that Word2Vec organizes behaviors from more general to more specific terms, revealing how behavior descriptions can range from broadly defined to more detailed and specific.

Despite the relatively strong alignment observed in the term frequency-based network, some behavior definitions were positioned closer to other categories than their manual labels would suggest. For example, definitions such as “Eating while walking,” “Grazing while standing,” and “Standing sleep (NREM, a state of non-rapid eye movement sleep associated with low activity and muscle tone)” appear near Moving Behaviors, likely due to the dominance of action-related terms such as “walking” or “standing.” Similarly, behaviors like “Lie active” and “Attempts of lying” cluster near movement-related definitions, reflecting shared lexical elements that imply motion or transition. These cases illustrate the semantic overlap that arises when behavior definitions incorporate features from more than one functional domain.

To enhance consistency and interpretability in ethological data, behavior definitions should be formulated with clearer and more specific semantic descriptions. Behaviors that involved overlapping or compound elements, such as multifunctional actions or vague descriptors, frequently appeared closer to categories that did not match their manual labels. Behaviors involving overlapping or compound elements, such as multifunctional actions or vague descriptors, are often clustered near categories that differed from their manual labels. For example, the definition “eating while walking” was grouped closer to movement-related behaviors rather than feeding, likely due to the inclusion of movement-related terms. These misalignments highlight how imprecise or overly broad language can introduce ambiguity and reduce the consistency of both manual annotation and automated classification. In contrast, the TF-IDF-based network showed that definitions using more distinctive and specific vocabulary tended to align better with human categorizations. Similarly, the radial structure of the Word2Vec-based network emphasized a continuum of semantic specificity, in which general or underspecified behaviors appeared near the center, while more precisely defined behaviors were positioned toward the periphery. These findings collectively point to the importance of linguistic clarity in shaping semantic structure. To minimize ambiguity, behavior definitions should clearly convey posture, context, and intent. For instance, lying ventrally may be described as “The animal’s abdomen is in full contact with the ground, limbs tucked beneath the body, and head resting on the ground,” while lying laterally may be defined as “The animal lies on its side, with one or both limbs extended and head in contact with the ground.” Definitions constructed in this way support more consistent manual labeling and improve the performance of automated clustering models. Ultimately, greater semantic precision enables the development of ethograms that are both interpretable and scalable across studies.

### 3.4. Practical Example of Image-Based Annotation Using Structured Behavior Definitions in LLMs

**Figure 6:**
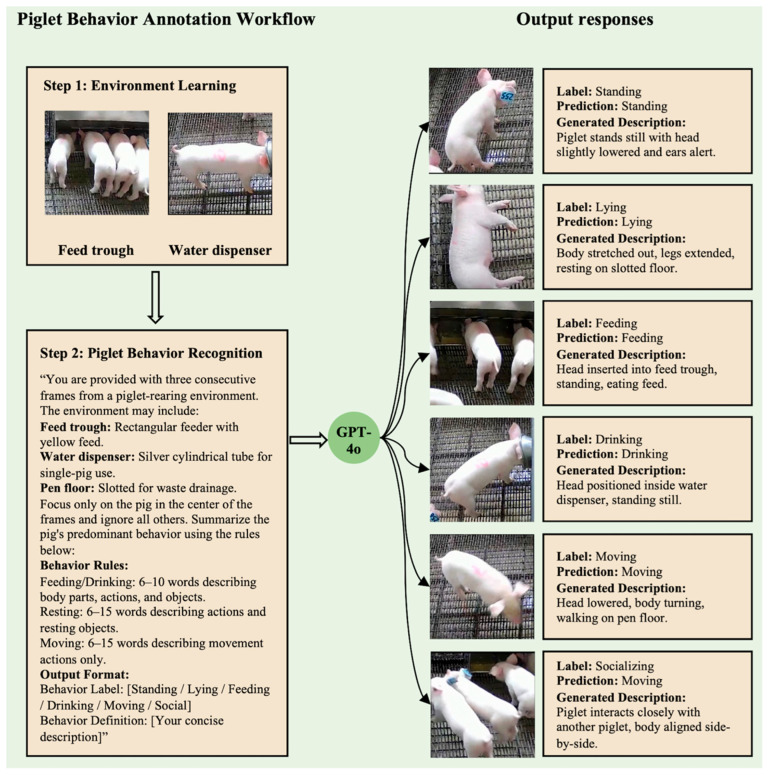
GPT-4o-based piglet behavior annotation results.

Based on the findings from the preceding semantic clustering analysis, which revealed that behavior definitions using precise and context-specific language aligned more closely with human-assigned categories, an image-based annotation experiment was conducted to evaluate the effect of structured semantic input on visual behavior recognition, as shown in Figure 6. The objective was to investigate whether providing a multimodal model with semantically structured behavior definitions would improve its ability to interpret and classify pig behaviors from still images. The focus was not only on annotation accuracy, but also on examining whether prompts containing clearly defined linguistic components, such as body part, behavioral actions, and contextual elements, could guide the model to generate predictions more consistent with expert annotations. To enable direct comparison, samples from the dataset introduced by [43] were used. In that study, behavioral annotations were assigned without the use of structured semantic definitions, allowing the current approach to be evaluated in relation to an existing non-structured method.

The experiment was implemented using ChatGPT-4o, a vision-language model capable of processing both textual and visual inputs. All 18 samples from the reference dataset were used, each consisting of three consecutive frames depicting a single pig. These samples were evenly distributed across six behavioral categories: standing, feeding, drinking, lying, moving, and socializing. All frame sets were selected to ensure that the target behavior was clearly visible and contextually unambiguous within the given frames. Structured prompts were introduced as the experimental variable and developed based on the results of the semantic structure analysis (see Figure 4). Feeding and drinking behaviors were described with 6 to 10 words, focusing on body parts, actions, and objects. Resting behaviors used 6 to 15 words, emphasizing actions and resting objects, while moving behaviors were described with 6 to 15 words, focusing on actions and locomotion. Prior to the annotation task, the model was provided with two reference images, one showing a feed trough and the other a water dispenser, and it was guided to recognize these objects. This step was intended to help the model identify contexts related to feeding and drinking in the subsequent annotation process.

To evaluate the effectiveness of the structured prompts, all 18 samples were submitted in three separate rounds of querying using the same set of prompts. The repeated querying was intended to reduce randomness in the model’s responses. In each round, the predicted label for each sample was compared to the ground-truth annotation, which had been independently assigned by two trained observers with complete agreement. A semantic correspondence score of 1 was assigned for each correct prediction and 0 for each incorrect one, yielding a raw score between 0 and 18 per round. Each raw score was then linearly rescaled to a 5-point scale, where 5 indicated perfect alignment with the ground truth and 0 indicated no alignment. The three normalized scores were used to assess the overall performance of the structured input approach. This allowed for a direct comparison with the non-structured annotation results available in the reference dataset. The 18 sample images are shown in Figure S1, and the model outputs are presented in Table S1.

Across three rounds of annotation, high and consistent semantic correspondence scores were achieved by ChatGPT-4o using structured input prompts. For each round, all 18 samples were annotated using the same prompt set, resulting in 17, 16, and 16 correct predictions, respectively. These raw scores were normalized to a five-point scale, yielding scores of 4.72, 4.44, and 4.44. The average score across rounds was 4.53, indicating that the model’s predictions were closely aligned with expert annotations. These results contrast with the score of 1.94 reported by [43], where non-structured definitions were used on the same dataset. Although the experimental conditions were not identical, the substantial difference in output alignment suggests that structured semantic input may offer practical advantages in behavior annotation tasks.

The structured prompts likely supported model performance by presenting behavior definitions in a format that emphasized core semantic components rather than relying on long and undifferentiated textual descriptions. By explicitly separating elements such as body parts, actions, and contextual objects, the prompts may have helped the model associate linguistic cues more directly with visual features. This structural clarity may have contributed to a more precise interpretation of static images by guiding the model to focus on specific behavioral attributes rather than inferring meaning from generalized or ambiguous phrasing. Although there is no established standard for what constitutes an optimal behavior definition, prediction consistency relative to expert-labeled annotations was used here as a practical indicator of definition effectiveness. In this context, definitions that resulted in model predictions more closely aligned with the ground-truth labels were considered more effective in supporting behavior recognition.

Despite these challenges, the structured prompt design revealed that behaviors such as moving and socializing are not devoid of identifiable features. As indicated by the semantic structure analysis results in Figure 4, 31.39% of the definitions associated with moving behavior included explicit action-related descriptions, suggesting the potential for more precise representation through quantifiable attributes. These may include displacement direction, the magnitude of movement, and changes in relative position, which are often detectable even in static frames. Similarly, although socializing behavior exhibits considerable variability and often depends on situational context, it can also be characterized using structured semantic components. Interaction types such as body alignment, facial orientation, proximity distance, and repeated attention cues may serve as latent indicators of social engagement. If such elements are systematically incorporated into behavior prompts, either through manual annotation guidelines or through automated detection strategies, models may be better guided to attend to behaviorally relevant visual cues.

These findings suggest that structured prompts can support the development of behavior definitions with clearer semantic structure and more informative descriptive features. Rather than relying on vague or inconsistent phrasing, such prompts guide the use of consistent terminology and relevant contextual elements. This approach has the potential to enhance the interpretability of automated behavior recognition models and support the integration of vision and language systems in livestock behavior analysis.

## 4. Limitation and Future Works

This study focused on four representative livestock species: cattle, sheep, pigs, and horses, selected for their shared anatomical and locomotive characteristics. Poultry were excluded due to their distinct physiological traits. Future research will aim to include poultry and other livestock species, providing a more comprehensive understanding of behavioral patterns across animals. This study concentrated on four core behaviors: feeding, drinking, resting, and moving. The classification of ‘moving behavior’ was broad, overlooking important details such as exploratory and social behaviors, including common forms such as aggression and mating [44,45]. Exploratory behavior, in particular, plays a key role in brain development and has been suggested to be influenced by various factors [46]. These behaviors involve complex, context-dependent movements and are difficult to classify consistently. Future research should refine these categories to improve the specificity of behavior classification and enhance the applicability of automated behavior recognition systems in livestock monitoring.

During the semantic clustering of behavior definitions, notable challenges were encountered due to the limitations of traditional text representation methods, such as term frequency and inverse document frequency, which struggled to capture nuanced semantic differences between behaviorally distinct but lexically similar verbs. For instance, definitions describing eating and drinking often share overlapping vocabulary with those involving movement, leading to incorrect groupings. These issues were compounded by variability in sentence structure, vocabulary usage, and descriptive detail across the dataset. Although a preprocessing pipeline was implemented, which included text normalization, lemmatization, and term filtering to improve data consistency, semantic ambiguity remained. To address this, future work will explore context-aware language models such as BERT [47] and BioBERT [48], which interpret word meaning in relation to its surrounding text and are more capable of distinguishing subtle behavioral differences. Incorporating domain knowledge, such as ethograms or species-specific behavioral hierarchies, may further guide clustering toward more biologically coherent and interpretable classifications.

Given the increasing application of computer vision in livestock behavior analysis and the current limitations in model interpretability, future research will aim to integrate traditional ethological understanding with computational approaches to enhance both accuracy and transparency in automated behavior recognition. To support this goal, the concept of digital definitions should be further developed. These are structured computational representations of animal behavior that formalize key semantic components such as body parts, spatial movement, and object interaction into measurable visual parameters. By translating ethological descriptions into quantifiable features, digital definitions offer a framework for linking language-based prompts with visual detection outputs in a consistent and interpretable manner. This approach offers potential for advancing real-time behavior monitoring and scalable annotation systems, thereby contributing to the broader goals of precision livestock farming and automated animal welfare assessment.

## 5. Conclusions

This study provides an in-depth analysis of collected behavioral definitions using both semantic clustering and manual classification methods, ultimately categorizing livestock behaviors into three main types: feeding and drinking behaviors, resting behaviors, and moving behaviors. While semantic clustering provided initial insights into textual patterns, its effectiveness was limited by overlapping verb meanings and variability in definition structure. As a result, manual classification was ultimately adopted to ensure accurate and consistent categorization. The results indicate that feeding and drinking behaviors tend to be described concisely, typically using 6 to 10 words, while resting and moving behaviors require more complex expressions, generally ranging from 6 to 15 words. Semantic structure analysis further revealed distinct compositional elements: feeding and drinking behaviors emphasize body parts, actions, and associated objects; resting behaviors highlight posture and environmental context; and moving behaviors are predominantly centered on action descriptors.

Based on these distinctions, structured definitions were used to construct prompts for a multimodal vision-language model to assess its capacity for image-based behavior annotation. The model achieved an average semantic correspondence score of 4.53 out of 5 across three rounds of prediction, demonstrating strong alignment with expert annotations. These results suggest that structured prompts informed by semantic analysis can enhance the interpretability and reliability of model outputs in behavior recognition tasks. These findings underscore the effectiveness of linguistically structured prompts in improving model alignment with expert annotations, offering a clear path toward more accurate, interpretable, and scalable behavior recognition systems. This approach has the potential to transform behavior recognition tasks, enabling more reliable and adaptable systems for real-world applications such as livestock monitoring and animal welfare management.

## Figures and Tables

**Table 1:** Criteria for selecting animal behavior definitions.

Behavior Category	Description
Feeding and Drinking Behavior	Behaviors related to eating or drinking, such as “Eat’’, “Drink’’, “Feed’’, “Graze’’, “Forage’’, etc.
Resting Behavior	Behaviors indicating rest or inactivity, such as “Lie’’, “Rest’’, “Sleep’’, “Inactive’’, etc.
Moving Behavior	Behaviors involving movement or activity, such as “Walk’’, “Run’’, “Turn’’, “Jump’’, “Trot’’, “Canter’’, “Move’’, “Play’’, “Active’’, “Locomotion’’, etc.

**Table 2:** Classification criteria for animal behavior definitions.

Semantic Category	Description
Subject	Includes animal nouns within the definition, such as “pigs”, “piglets”, “horse”, “sheep”, “cattle”, etc.
Body Parts	Refers to nouns describing parts of the animal’s body, such as “feet”, “mouth”, “belly”, etc.
Action or Verb	Includes verbs that indicate actions performed by the animal, such as “walk”, “run”, “graze”, etc.
Location or Object	Specifies where the action occurs or the object involved, such as “ground”, “in feeder”, “in trough”, etc.
Time-related Description	Refers to time-related details specifying the duration of the action, such as “3 s”, “5 s”, etc.
Action-related Description	Describes the intensity of the action, such as “slow”, “rapid”, etc.
Distance-related Description	Indicates the range of movement, such as “5 cm”, “15 cm”, etc.

## Data Availability

The data that support the findings of this study are available upon reasonable request.

## Supplementary Materials

The following supporting information can be downloaded at: https://www.mdpi.com/article/10.3390/ani15203030/s1, Figure S1: Example Piglet Behavior Images for Image-Based Annotation; Table S1: Behavior Definitions and Labels Generated by ChatGPT-4o. Table S2. Sources of the Dataset References.
