# Supplementary material for: Foundations of Livestock Behavioral Recognition: Ethogram Analysis of Behavioral Definitions and Its Practices in Multimodal Large Language Models
Source: Animals (Basel). 2025 Oct 19;15(20):3030. doi: 10.3390/ani15203030 (PMC12561510; doi:10.3390/ani15203030)
Supplement: Supplementary file 1 [file animals-15-03030-s001.zip › animals-3887865-supplementary.pdf]

Supplementary Materials

Supplementary I Piglet Behavior Dataset Images

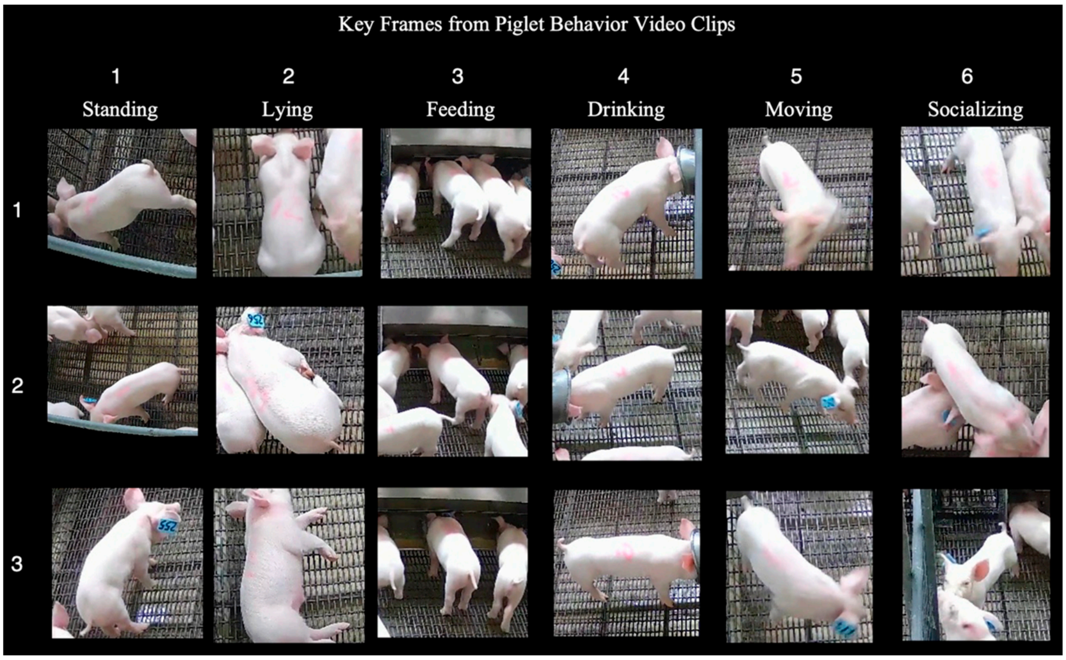

Fig. S1. Example Piglet Behavior Images for Image-Based Annotation.

Supplementary II Behavior Definitions and Labels Generated by ChatGPT-4o

Table S1

Behavior definitions and labels generated by ChatGPT-4o.

Behavior	Sample ID	Definition 1	Definition 2	Definition 3
Standing	1-1	Piglet stretches its legs, shifts body slightly forward. **(Label: Moving)**	Piglet steps forward with head lowered and body shifting. **(Label: Moving)**	Head and body shifting position, legs extended on floor. **(Label: Moving)**
	1-2	Piglet stands still with head lowered toward the ground. **(Label: Standing)**	Piglet stands still with head lowered toward the ground. **(Label: Standing)**	Piglet's body shifts forward with slight leg movements on slotted floor. **(Label: Moving)**
	1-3	Piglet stands still with its head raised slightly upward. **(Label: Standing)**	Piglet stands still with head slightly lowered and ears alert. **(Label: Standing)**	Piglet stands still with head slightly lowered and ears alert. **(Label: Standing)**

Lying	2-1	Piglet lies motionless on slotted pen floor. **(Label: Lying)**	Piglet lies still on slotted pen floor, body fully relaxed. **(Label: Lying)**	Body stretched out, legs relaxed on slotted pen floor. **(Label: Lying)**
	2-2	Piglet lies on its side, body fully relaxed against another piglet. **(Label: Lying)**	Piglet lies on its side, body relaxed, next to another piglet. **(Label: Lying)**	Body lying on slotted floor, head resting, minimal movement. **(Label: Lying)**
	2-3	Piglet lies still on its side on slotted pen floor. **(Label: Lying)**	Piglet lies on its side with body fully relaxed. **(Label: Lying)**	Body stretched out, legs extended, resting on slotted floor. **(Label: Lying)**
Feeding	3-1	Piglet stands with head lowered into feed trough, eating feed. **(Label: Feeding)**	Head and mouth interacting with the feed trough. **(Label: Feeding)**	Head inserted into trough, eating from yellow feed. **(Label: Feeding)**
	3-2	Piglet stands still with its head lowered into the feed trough, actively eating feed. **(Label: Feeding)**	Head lowered into the feed trough, standing still. **(Label: Feeding)**	Head lowered, mouth interacting with the rectangular feed trough. **(Label: Feeding)**
	3-3	Piglet stands with head inserted into the feed trough, eating feed. **(Label: Feeding)**	Head lowered, mouth interacting with feed trough. **(Label: Feeding)**	Head inserted into feed trough, standing, eating feed. **(Label: Feeding)**
Drinking	4-1	Piglet stands with mouth interacting with water dispenser. **(Label: Drinking)**	Head positioned inside water dispenser, standing still. **(Label: Drinking)**	Head inside water dispenser, body standing still, interacting with silver tube. **(Label: Drinking)**
	4-2	Piglet stands with mouth interacting with the water dispenser tube. **(Label: Drinking)**	Head extended into silver cylindrical water dispenser. **(Label: Drinking)**	Head lowered, mouth interacting with silver cylindrical water tube. **(Label: Drinking)**

	4-3	Piglet stands with head inserted into the water dispenser. **(Label: Drinking)**	Head lowered, mouth interacting with silver water dispenser. **(Label: Drinking)**	Head lowered, mouth interacting with water dispenser tube. **(Label: Drinking)**
	5-1	Piglet moves forward with head lowered and legs stepping. **(Label: Moving)**	Head lowered, body turning, walking on pen floor. **(Label: Moving)**	Pig walking forward with lowered head, exploring pen floor. **(Label: Moving)**
Moving	5-2	Piglet steps forward with head lowered and legs shifting. **(Label: Moving)**	Head and legs shifting, piglet stepping forward slightly. **(Label: Moving)**	Piglet walking forward, legs lifted, head low. **(Label: Moving)**
	5-3	Piglet walks forward with head lowered, body shifting direction. **(Label: Moving)**	Piglet moves forward with head lowered, stepping across the pen. **(Label: Moving)**	Pig's body upright, legs moving forward across slotted floor. **(Label: Moving)**
	6-1	Piglet interacts closely with another piglet, body aligned side-by-side. **(Label: Socializing)**	Head lowered, walking forward on slotted pen floor. **(Label: Moving)**	Two piglets standing close, head-to-head sniffing interaction. **(Label: Socializing)**
Socializing	6-2	Piglet climbs over another piglet while interacting physically. **(Label: Socializing)**	Piglet climbs over another piglet's back. **(Label: Socializing)**	Body pressed against other pig, moving forward. **(Label: Socializing)**
	6-3	Piglet faces and interacts with another piglet near the enclosure wall. **(Label: Socializing)**	Piglet faces and interacts with another piglet near the enclosure. **(Label: Socializing)**	Head close to other piglet, interacting actively. **(Label: Socializing)**

Appendix III Sources of the Dataset References

Table S2

Sources of the Dataset References.

Title	Year	Journal
Behavioural changes in weaned piglets orally challenged with Escherichia coli F4 and supplemented with in-feed protected acid salts	2023	Applied Animal Behaviour Science

Effects over time of different types of sounds on gestating sows' behavior	2023	Applied Animal Behaviour Science
Usage of outdoor runs and defaecation behaviour of fattening pigs	2023	Applied Animal Behaviour Science
The effects of stocking density on behavior and biological functioning of penned sheep under continuous heat load conditions.	2023	Journal of Animal Science
Behavioral responses of horses (Equus ferus caballus) vary in response to potentially threatening odor condition and aversive social odor stimuli	2023	Applied Animal Behaviour Science
Administration of procaine-based local anaesthetic prior to surgical castration influences post-operative behaviours of piglets	2023	Applied Animal Behaviour Science
Effects of a competitive feeding situation on the behaviour and energy requirements of gestating sows	2023	Applied Animal Behaviour Science
Validation of non-invasive sensor technologies to measure interaction with enrichment material in weaned fattening pigs	2023	Applied Animal Behaviour Science
Play behaviour positively relates to weight gain, feeding behaviour and drinking behaviour in weaner pigs (Sus scrofa)	2023	Applied Animal Behaviour Science
Pre-weaning social behaviours and peripheral serotonin levels are associated with behavioural and physiological responses to weaning and social mixing in pigs	2023	Applied Animal Behaviour Science
Rub 'n' roll-Pigs, Sus scrofa domesticus, display rubbing and rolling behaviour when exposed to odours	2023	Applied Animal Behaviour Science
Classifying the posture and activity of ewes and lambs using accelerometers and machine learning on a commercial flock	2022	Applied Animal Behaviour Science
Impact of enhanced compared to restricted milk feeding on the behaviour and health of organic dairy calves	2022	Applied Animal Behaviour Science
Impact of two commercial weaning and transport strategies on piglet behaviour, body weight change, lesions and lameness following transport	2022	Applied Animal Behaviour Science
Refined methodology for identification of bitterness aversion in mature rams through quantification of fluid intake and behavioral response to phenylthiocarbamide	2022	Applied Animal Behaviour Science
Single- and multi-species groups: A descriptive study of cattle and broiler behaviour on pasture	2022	Applied Animal Behaviour Science
Sow behavior during introduction to a large dynamic group is influenced by familiarity and method	2022	Applied Animal Behaviour Science
Long-duration transit and food and water deprivation alter behavioral activities and aggressive interactions at the feed bunk in beef feedlot steers	2022	Journal of Animal Science
Effect of bupivacaine liposome suspension administered as a local anesthetic block on indicators of pain and distress during and after surgical castration in dairy calves	2022	Journal of Animal Science
Does the duration of repeated temporary separation affect welfare in dairy cow-calf contact systems?	2022	Applied Animal Behaviour Science
Effect of reduced dietary protein level on finishing pigs' harmful social behaviour before and after an abrupt dietary change	2022	Applied Animal Behaviour Science
Effects of farrowing hut design on maternal and thermoregulatory behaviour in outdoor housed sows and piglets	2022	Applied Animal Behaviour Science
Effects of physical enrichment and pair housing before weaning on growth, behaviour and cognitive ability of calves after weaning and regrouping	2022	Applied Animal Behaviour Science
Estimating the optimal number of sampling days and patterns for recording calf behaviours in pre-weaning dairy calves	2022	Applied Animal Behaviour Science
Evaluating the utility of a CO2 surgical laser for piglet tail docking to reduce behavioral and physiological indicators of pain and to improve wound healing: A pilot study	2022	Applied Animal Behaviour Science
Estimating the repeatability of behavioral traits in Norduz sheep subjected to an arena test	2022	Applied Animal Behaviour Science

Acute changes in oxytocin predict behavioral responses to foundation training in horses	2022	Applied Animal Behaviour Science
Access to chewable materials during lactation affects sow behaviour and interaction with piglets	2021	Applied Animal Behaviour Science
Hourly activity patterns and behaviour-based management of feedlot steers with and without a cattle brush	2021	Applied Animal Behaviour Science
Strategic use of straw as environmental enrichment for prepartum sows in farrowing crates	2021	Applied Animal Behaviour Science
What type of loafing areas do housed dairy cattle prefer?	2021	Applied Animal Behaviour Science
Varied diets: implications for lamb performance, rumen characteristics, total antioxidant status, and welfare	2021	Journal of Animal Science
Evaluation of sow thermal preference across three stages of reproduction	2021	Journal of Animal Science
Behavioral and stress responses to feeding time in pregnant sows under limit-fed regime	2021	Journal of Animal Science
Exploring stable-based behaviour and behaviour switching for the detection of bilateral pain in equines	2021	Applied Animal Behaviour Science
Horses' physiological and behavioural responses during undergraduate veterinary practical teaching classes	2021	Applied Animal Behaviour Science
Behavior and body temperature alterations in piglets anesthetized for castration during a four-hour recovery phase	2021	Applied Animal Behaviour Science
Impact of paper bedding on lying behaviour and welfare in lactating dairy cows	2021	Applied Animal Behaviour Science
Keeping littermates together instead of social mixing benefits pig social behaviour and growth post-weaning	2021	Applied Animal Behaviour Science
Lying posture does not accurately indicate sleep stage in dairy cows	2021	Applied Animal Behaviour Science
Rooting for feed: Mixing corn pellets into rooting material tends to increase the presence of grower and finisher pigs in the rooting area but not its cleanliness	2021	Applied Animal Behaviour Science
Subjective methods of quantifying temperament in heifers are indicative of physiological stress	2021	Applied Animal Behaviour Science
Tail-biting behaviour pre-weaning: Association between other pig-directed and general behaviour in piglets	2021	Applied Animal Behaviour Science
Technical contribution: use of continuous recording video monitoring of maintenance and pain behaviors in piglets after surgical castration to validate six continuous time sampling periods for behavior scoring	2021	Journal of Animal Science
The effect of altering routine husbandry factors on sleep duration and memory consolidation in the horse	2021	Applied Animal Behaviour Science
Effects of physical enrichment items and social housing on calves' growth, behaviour and response to novelty	2021	Applied Animal Behaviour Science
Time budgets of group-housed pigs in relation to social aggression and production	2021	Journal of Animal Science
The effect of free-stall versus strawyard housing and access to pasture on dairy cow locomotor activity and time budget	2020	Applied Animal Behaviour Science
Impact of a cattle brush on feedlot steer behavior, productivity and stress physiology	2020	Applied Animal Behaviour Science
Maize silage as enrichment material improves the welfare of growing-finishing pigs in environmentally-friendly pens	2020	Applied Animal Behaviour Science
The opening of a hinged farrowing crate improves lactating sows' welfare	2020	Applied Animal Behaviour Science
Early life thermal stress: impacts on future temperature preference in weaned pigs (3 to 15 kg)	2020	Journal of Animal Science
Evaluation and mitigation of the effects of in utero heat stress on piglet growth performance, postabsorptive metabolism, and stress response following weaning and transport	2020	Journal of Animal Science
Behavioral and cortisol responses to feeding frequency in pregnant sows under isocaloric intake	2020	Journal of Animal Science
Effects of grazing muzzles on behavior and physiological stress of individually housed grazing miniature horses	2020	Applied Animal Behaviour Science

Effects of grazing muzzles on behavior, voluntary exercise, and physiological stress of miniature horses housed in a herd	2020	Applied Animal Behaviour Science
Effect of analgesic strategies on pain behaviour associated with combined ring castration and hot iron tail docking in Merino lambs	2020	Applied Animal Behaviour Science
Pre-weaning socialization and environmental enrichment affect life-long response to regrouping in commercially-reared pigs	2020	Applied Animal Behaviour Science
Qualitative behavioural assessment of pain in castrated lambs	2020	Applied Animal Behaviour Science
Evaluating the utility of a CO2 surgical laser for piglet castration to reduce pain and improve wound healing: a pilot study	2020	Journal of Animal Science
Which types of rooting material give weaner pigs most pleasure?	2020	Applied Animal Behaviour Science
Palatability assessment in horses in relation to lateralization and temperament	2020	Applied Animal Behaviour Science
A single dose of ketoprofen in the immediate postpartum period has the potential to improve dairy calf welfare in the first 48 h of life	2019	Applied Animal Behaviour Science
Technical note: validation of a system for monitoring individual behavior in beef heifers	2019	Journal of Animal Science
Replacing dietary antibiotics with 0.20% L-glutamine in swine nursery diets: impact on health and productivity of pigs following weaning and transport	2019	Journal of Animal Science
How many pigs within a group need to be sick to lead to a diagnostic change in the group's behavior?	2019	Journal of Animal Science
Development of scoring systems for abnormal rising and lying down by dairy cattle, and their relationship with other welfare outcome measures	2019	Applied Animal Behaviour Science
Does dairy calves' motivation for social play behaviour build up over time?	2019	Applied Animal Behaviour Science
Effects of flooring surface and a supplemental heat source on location preference, behaviour and growth rates of dairy goat kids	2019	Applied Animal Behaviour Science
Implication and impact of straw provision on behaviour, lesions and pen hygiene on commercial farms rearing undocked pigs	2019	Applied Animal Behaviour Science
Playful pigs: early life play-fighting experience influences later life contest dynamics	2019	Animal Behaviour
The effect of blankets on horse behaviour and preference for shelter in Nordic winter conditions	2019	Applied Animal Behaviour Science
Behaviour and physiology of sheep exposed to ammonia at a similar concentration to those experienced by sheep during export by sea	2018	Applied Animal Behaviour Science
Dietary diversity affects feeding behaviour of suckling piglets	2018	Applied Animal Behaviour Science
Evaluation of alternatives to cautery disbudding of dairy goat kids using behavioural measures of post-treatment pain	2018	Applied Animal Behaviour Science
Inducing positive emotions: Behavioural and cardiac responses to human and brushing in ewes selected for high vs low social reactivity	2018	Applied Animal Behaviour Science
The sickness response at and before clinical diagnosis of spontaneous bovine respiratory disease	2018	Applied Animal Behaviour Science
Tail posture predicts tail biting outbreaks at pen level in weaner pigs	2018	Applied Animal Behaviour Science
"Hay-bags" and "Slow feeders": Testing their impact on horse behaviour and welfare	2018	Applied Animal Behaviour Science
Can body nosing in artificially reared piglets be reduced by sucking and massaging dummies?	2018	Applied Animal Behaviour Science
Dairy goats use outdoor runs of high quality more regardless of the quality of indoor housing	2018	Applied Animal Behaviour Science
Early indicators of tail biting outbreaks in pigs	2018	Applied Animal Behaviour Science

Effects of space allowance and simulated sea transport motion on behavioural and physiological responses of sheep	2018	Applied Animal Behaviour Science
Nest-building behaviour and activity budgets of sows provided with different materials	2018	Applied Animal Behaviour Science
Behavioural and physiological responses to clove oil injected under the horn bud of calves	2018	Applied Animal Behaviour Science
Playful pigs: Evidence of consistency and change in play depending on litter and developmental stage	2018	Applied Animal Behaviour Science
Pre-weaning environmental enrichment increases piglets' object play behaviour on a large scale commercial pig farm	2018	Applied Animal Behaviour Science
The sex ratio of a litter affects the behaviour of its female pigs until at least 16 weeks of age	2018	Applied Animal Behaviour Science
Behavior changes associated with lameness in sows	2017	Applied Animal Behaviour Science
Behaviour of White Fulani calves grazing Panicum/Stylo pasture in Southwest Nigeria	2017	Applied Animal Behaviour Science
Behavioural response of dairy goat kids to cautery disbudding	2017	Applied Animal Behaviour Science
Confinement of sows 24 h before expected farrowing affects the performance of nest building behaviours but not progress of parturition	2017	Applied Animal Behaviour Science
Daytime summer access to pasture vs. free-stall barn in dairy cows with year-long outdoor experience: A case study	2017	Applied Animal Behaviour Science
The effect of social dynamics and environment at time of early weaning on short- and long-term lamb behavior in a pasture and feedlot setting	2017	Applied Animal Behaviour Science
Emotional states and emotional contagion in pigs after exposure to a positive and negative treatment	2017	Applied Animal Behaviour Science
Hiding walls for fattening pigs: Do they affect behavior and performance?	2017	Applied Animal Behaviour Science
Impact of prenatal stress and environmental enrichment prior to weaning on activity and social behaviour of piglets (Sus scrofa)	2017	Applied Animal Behaviour Science
Influence of enrichment material and herbal compounds in the behaviour and performance of growing pigs	2017	Applied Animal Behaviour Science
A novel method for the analysis of social structure allows in-depth analysis of sow rank in newly grouped sows	2017	Applied Animal Behaviour Science
Effect of timing of subcutaneous meloxicam administration on indicators of pain after knife castration of weaned calves	2017	Journal of Animal Science
Technical note: Instantaneous sampling intervals validated from continuous video observation for behavioral recording of feedlot lambs	2017	Journal of Animal Science
Effect of band and knife castration of beef calves on welfare indicators of pain at three relevant industry ages: I. Acute pain	2017	Journal of Animal Science
Barren diets increase wakeful inactivity in calves	2017	Applied Animal Behaviour Science
Gradual weaning during an extended lactation period improves performance and behavior of pigs raised in a multi-suckling system	2017	Applied Animal Behaviour Science
An improved design for the spatial judgement task in domestic pigs	2017	Applied Animal Behaviour Science
Self-administration by consumption of flunixin in feed alleviates the pain and inflammation associated with castration and tail docking of lambs	2017	Applied Animal Behaviour Science
Effect of social feeding environment on the feeding behaviour of dairy cows and their willingness to consume a novel feed	2016	Applied Animal Behaviour Science
Long-term effects of straw blocks in pens with finishing pigs and the interaction with boar type	2016	Applied Animal Behaviour Science
Stocking density affects welfare indicators of growing pigs of different group sizes after regrouping	2016	Applied Animal Behaviour Science
A synthetic olfactory agonist reduces aggression when sows are mixed into small groups	2016	Applied Animal Behaviour Science

Fever, feeding, and grooming behavior around peak clinical signs in bovine respiratory disease	2016	Journal of Animal Science
Including dietary fiber and resistant starch to increase satiety and reduce aggression in gestating sows	2016	Journal of Animal Science
Group and individual sow behavior is altered in early gestation by space allowance in the days immediately following grouping	2016	Journal of Animal Science
Pain alleviation during castration of piglets: a comparative study of different farm options	2016	Journal of Animal Science
Does housing influence maternal behaviour in sows?	2016	Applied Animal Behaviour Science
Complex contests and the influence of aggressiveness in pigs	2016	Animal Behaviour
The effect of nursing on the cow-calf bond	2015	Applied Animal Behaviour Science
Effects of sounds of different quality on the behaviour and heart beat parameters of goats	2015	Applied Animal Behaviour Science
Predicting time spent on different behavioural activities from physicochemical properties of fibrous diets in finishing pigs	2015	Applied Animal Behaviour Science
Straw applications in growing pigs: Effects on behavior, straw use and growth	2015	Applied Animal Behaviour Science
Welfare outcomes for 3-and 6-month-old beef calves in a tropical environment castrated surgically or by applying rubber rings	2015	Applied Animal Behaviour Science
The effects of different flooring types on the behavior, health, and welfare of finishing beef steers	2015	Journal of Animal Science
The effect of presenting forage in multi-layered haynets and at multiple sites on night time budgets of stabled horses	2015	Applied Animal Behaviour Science
The effects of a high-starch or high-fibre diet on equine reactivity and handling behaviour	2015	Applied Animal Behaviour Science
Daytime shelter use of individually kept horses during Swedish summer	2015	Journal of Animal Science
Dairy calves' preference for rearing substrate	2015	Applied Animal Behaviour Science
Evidence for litter differences in play behaviour in pre-weaned pigs	2015	Applied Animal Behaviour Science
The behaviour of gestating dairy ewes under different space allowances	2014	Applied Animal Behaviour Science
The effect of ketoprofen on post-partum behaviour in sows	2014	Applied Animal Behaviour Science
Effects of restraint with or without blinds at the feed barrier on feeding and agonistic behaviour in horned and hornless goats	2014	Applied Animal Behaviour Science
Tail biting behaviour and tail damage in pigs and the relationship with general behaviour: Predicting the inevitable?	2014	Applied Animal Behaviour Science
Effects of tail docking on behavior of confined feedlot cattle	2014	Journal of Animal Science
Effects of pellet diameter during and after lactation on feed intake of piglets pre- and postweaning	2014	Journal of Animal Science
Behavioral and physiological responses of primiparous sows to mixing with older, unfamiliar sows	2014	Journal of Animal Science
Agreement between different sleep states and behaviour indicators in dairy cows	2014	Applied Animal Behaviour Science
Can ear postures reliably measure the positive emotional state of cows?	2014	Applied Animal Behaviour Science
Does stocking density modify affective state in pigs as assessed by cognitive bias, behavioural and physiological parameters?	2014	Applied Animal Behaviour Science
Serotonin's role in piglet mortality and thriftiness	2014	Journal of Animal Science
Group space allowance has little effect on sow health, productivity, or welfare in a free-access stall system	2014	Journal of Animal Science
The effect of birth weight and age at tail docking and ear notching on the behavioral and physiological responses of piglets	2014	Journal of Animal Science
Effects of flow rate and gas mixture on the welfare of weaned and neonate pigs during gas euthanasia	2014	Journal of Animal Science

How much is enough? The amount of straw necessary to satisfy pigs' need to perform exploratory behaviour	2014	Applied Animal Behaviour Science
Investigating the motivation to play in lambs	2014	Applied Animal Behaviour Science
Rearing substrate and space allowance influences locomotor play behaviour of dairy calves in an arena test	2014	Applied Animal Behaviour Science
Responses to novel situations of female and castrated male pigs with divergent social breeding values and different backtest classifications in barren and straw-enriched housing	2014	Applied Animal Behaviour Science
The influence of floor type before and after 10 weeks of age on osteochondrosis in growing gilts	2014	Journal of Animal Science
Automatic registration of grazing behaviour in dairy cows using 3D activity loggers	2013	Applied Animal Behaviour Science
Does housing nulliparous dairy cows with multiparous animals prior to calving influence welfare-and production-related parameters after calving?	2013	Applied Animal Behaviour Science
Does the presence of a human affect the preference of enrichment items in young, isolated pigs?	2013	Applied Animal Behaviour Science
Effect of on farm mixing and slaughter strategy on behaviour, welfare and productivity in Duroc finished entire male pigs	2013	Applied Animal Behaviour Science
Effects of two substrate types on the behaviour, cleanliness and thermoregulation of dairy calves	2013	Applied Animal Behaviour Science
The importance of diet choice on stress-related responses by lambs	2013	Applied Animal Behaviour Science
Effect of group size on behavior, health, production, and welfare of veal calves	2013	Journal of Animal Science
Precalving temperament and maternal defensiveness are independent traits but precalving fear may impact calf growth	2013	Journal of Animal Science
Evaluation of the impacts of spaying by either the dropped ovary technique or ovariectomy via flank laparotomy on the welfare of Bos indicus beef heifers and cows	2013	Journal of Animal Science
Prenatal stress puzzle, the oxytocin piece: Prenatal stress alters the behaviour and autonomic regulation in piglets, insights from oxytocin	2013	Applied Animal Behaviour Science
Effects of tail docking and teeth clipping on the physiological responses, wounds, behavior, growth, and backfat depth of pigs	2013	Journal of Animal Science
Effects of neonatal castration on social behaviour, human–animal relationship and feeding activity in finishing pigs reared in a conventional or an enriched housing	2013	Applied Animal Behaviour Science
Backtest and novelty behavior of female and castrated male piglets, with diverging social breeding values for growth	2013	Journal of Animal Science
Behaviour and welfare of veal calves fed different amounts of solid feed supplemented to a milk replacer ration adjusted for similar growth	2012	Applied Animal Behaviour Science
Establishing the extent of behavioural reactions in dairy cattle to a leg mounted activity monitor	2012	Applied Animal Behaviour Science
Relationship between growth rate and oral manipulation, social nosing, and aggression in finishing pigs	2012	Applied Animal Behaviour Science
Space allowance for gestating sows in pens: Behavior and immunity	2012	Journal of Animal Science
A novel scale of behavioural indicators of stress for use with domestic horses	2012	Applied Animal Behaviour Science
Sleep in dairy cows recorded with a non-invasive EEG technique	2012	Applied Animal Behaviour Science
Identifying potential risk situations for humans when removing horses from groups	2012	Applied Animal Behaviour Science
A longitudinal study of the effects of providing straw at different stages of life on tail-biting and other behaviour in commercially housed pigs	2011	Applied Animal Behaviour Science
Peat as environmental enrichment for piglets: The effect on behaviour, skin lesions and production results	2011	Applied Animal Behaviour Science
Effects of enrichment items on activity and social interactions in domestic horses (Equus caballus)	2011	Applied Animal Behaviour Science

Object recognition and generalisation during habituation in horses	2011	Applied Animal Behaviour Science
Genetic and environmental effects on piglet survival and maternal behaviour of the farrowing sow	2011	Applied Animal Behaviour Science
Getting around social status: Motivation and enrichment use of dominant and subordinate sows in a group setting	2011	Applied Animal Behaviour Science
Nitrous oxide by itself is insufficient to relieve pain due to castration in piglets	2011	Journal of Animal Science
Behavior of piglets after castration with or without carbon dioxide anesthesia	2011	Journal of Animal Science
Effects of repeated regrouping on horse behaviour and injuries	2011	Applied Animal Behaviour Science
Behaviour of growing lambs housed on straw or woodchip bedding materials and their preference for floor type	2010	Applied Animal Behaviour Science
Effects of fermentable starch on behaviour of growing pigs in barren or enriched housing	2010	Applied Animal Behaviour Science
'Subordination style' in pigs? The response of pregnant sows to mixing stress affects their offspring's behaviour and stress reactivity	2010	Applied Animal Behaviour Science
The effect of dentition on feeding development in piglets and on their growth and behavior after weaning	2010	Journal of Animal Science
Behavior and peripheral amine concentrations in relation to ractopamine feeding, sex, and social rank of finishing pigs	2010	Journal of Animal Science
Effect of feed barrier design and feed space allowance on performance and behavioural parameters in dairy cows	2010	Applied Animal Behaviour Science
Reliability and feasibility of selected measures concerning resting behaviour for the on-farm welfare assessment in dairy cows	2010	Applied Animal Behaviour Science
Behaviour of badgers (Meles meles) in farm buildings: Opportunities for the transmission of Mycobacterium bovis to cattle?	2009	Applied Animal Behaviour Science
The role of novelty in environmental enrichment for the weaned pig	2009	Applied Animal Behaviour Science
The use of a mirror reduces isolation stress in horses being transported by trailer	2009	Applied Animal Behaviour Science
Measuring cutaneous thermal nociception in group-housed pigs using laser technique—Effects of laser power output	2009	Applied Animal Behaviour Science
Grouping horses according to gender—Effects on aggression, spacing and injuries	2009	Applied Animal Behaviour Science
Consistency and influence on performance of behavioural differences in Large White and Landrace purebred pigs	2009	Applied animal behaviour science
Influence of access to straw provided in racks on the welfare of sows in large dynamic groups	2008	Applied Animal Behaviour Science
The influence of season and of providing a water trough on stream use by beef cattle grazing hill-country in New Zealand	2008	Applied Animal Behaviour Science
Music during play-time: Using context conditioning as a tool to improve welfare in piglets	2008	Applied Animal Behaviour Science
Negative versus positive reinforcement: An evaluation of training strategies for rehabilitated horses	2008	Applied Animal Behaviour Science
Predator odour per se does not frighten domestic horses	2008	Applied Animal Behaviour Science
Influence of different types of environmental enrichment on the behaviour of finishing pigs in two different housing systems: 2. Ratio of pigs to enrichment	2007	Applied Animal Behaviour Science
Time budget-, behavioral synchrony- and body score development of a newly released Przewalski's horse group Equus ferus przewalskii, in the Great Gobi B strictly protected area in SW Mongolia	2007	Applied Animal Behaviour Science
Evaluation of a novel method of horse personality assessment: Rater-agreement and links to behaviour	2007	Applied Animal Behaviour Science
Announcing the arrival of enrichment increases play behaviour and reduces weaning-stress-induced behaviours of piglets directly after weaning	2006	Applied Animal Behaviour Science

The effect of age at introduction and number of milk-portions on the behaviour of group housed dairy calves fed by a computer controlled milk feeder	2006	Applied Animal Behaviour Science
Effect of temporary straw bedding on pigs' behaviour, performance, cortisol and meat quality	2006	Applied Animal Behaviour Science
Effects of rearing and housing environment on behaviour and performance of pigs with different coping characteristics	2006	Applied Animal Behaviour Science
Effects of species-relevant environmental enrichment on the behaviour and productivity of finishing pigs	2006	Applied Animal Behaviour Science
Preference of growing pigs for illuminance	2006	Applied Animal Behaviour Science
The accumulation of skin lesions and their use as a predictor of individual aggressiveness in pigs	2006	Applied Animal Behaviour Science
The effect of providing shredded paper or ropes to piglets in farrowing crates on their behaviour and health and the behaviour and health of their dams	2006	Applied Animal Behaviour Science
Does floor heating around parturition affect the vitality of piglets born to loose housed sows?	2006	Applied Animal Behaviour Science
Alternatives to nose-ringing in outdoor sows: the provision of root crops	2005	Applied Animal Behaviour Science
Designated rooting areas to reduce pasture damage by pregnant sows	2005	Applied Animal Behaviour Science
The effect of two piglet teeth resection procedures on the welfare of sows in farrowing crates. Part 2	2005	Applied Animal Behaviour Science
Responses of horses to novel visual, olfactory and auditory stimuli	2005	Applied Animal Behaviour Science
The short-term effects of increasing meal frequency on stereotypic behaviour of stabled horses	2005	Applied Animal Behaviour Science
The effect of two teeth resection procedures on the welfare of piglets in farrowing crates. Part 2	2005	Applied Animal Behaviour Science
Sensory varieties in concentrate diets for stabled horses: effects on behaviour and selection	2005	Applied Animal Behaviour Science
Assessing long-term behavioural effects of feeding motivation in group-housed pregnant sows; what, when and how to observe	2004	Applied Animal Behaviour Science
Behavioural responses of dairy cows toward novel stimuli presented in the home environment	2004	Applied Animal Behaviour Science
Alternatives to nose-ringing in outdoor sows: 1. The provision of a sacrificial rooting area	2003	Applied Animal Behaviour Science
Behavioural and adrenocortical responses of dairy cows toward novel food: effects of food deprivation, milking frequency and energy density in the daily ration	2003	Applied Animal Behaviour Science
Assessment of pain induced by castration in piglets: behavioral and physiological responses over the subsequent 5 days	2003	Applied Animal Behaviour Science
Lying characteristics as determinants for space requirements in pigs	2003	Applied Animal Behaviour Science
Pre-mixing as a technique for facilitating subgroup formation and reducing sow aggression in large dynamic groups	2003	Applied Animal Behaviour Science
A systematic approach towards developing environmental enrichment for pigs	2003	Applied Animal Behaviour Science
Sow behaviour involved in the crushing of piglets in outdoor farrowing huts—a brief report	2003	Applied Animal Behaviour Science
Analysis of belly nosing and associated behaviour among pigs weaned at 12–14 days of age	2002	Applied Animal Behaviour Science
Behaviour of two genotypes of growing–finishing pig in three different housing systems	2002	Applied Animal Behaviour Science
The separate and interactive effects of handling and environmental enrichment on the behaviour and welfare of growing pigs	2002	Applied Animal Behaviour Science
Influence of housing on weaning horse behavior and subsequent welfare	2002	Applied Animal Behaviour Science
Savaging gilts are more restless and more responsive to piglets during the expulsive phase of parturition	2002	Applied Animal Behaviour Science

How reliable is temperament assessment in the domestic horse (Equus caballus)?	2002	Applied Animal Behaviour Science
Influence of environmental temperature on PGF2α-induced nest building in female pigs	2001	Applied Animal Behaviour Science
PGF2α-induced nest building and choice behaviour in female domestic pigs	2001	Applied Animal Behaviour Science
Activity, oral behaviour and slaughter data as welfare indicators in veal calves: a comparison of three housing systems	2001	Applied Animal Behaviour Science
Behaviour of calves at introduction to nurse cows after the colostrum period	2001	Applied Animal Behaviour Science
The effect of parity and environmental restriction on behavioural and physiological responses of pre-parturient pigs	2001	Applied Animal Behaviour Science
Deprivation of straw bedding alters PGF2α-induced nesting behaviour in female pigs	2000	Applied Animal Behaviour Science
Effects of handling aids on calf behavior	2000	Applied Animal Behaviour Science
